# When in doubt follow your nose—a wayfinding strategy

**DOI:** 10.3389/fpsyg.2014.01363

**Published:** 2014-11-26

**Authors:** Tobias Meilinger, Julia Frankenstein, Heinrich H. Bülthoff

**Affiliations:** ^1^Max Planck Institute for Biological Cybernetics, Tübingen, Germany; ^2^Center for Cognitive Science, University of Freiburg, Freiburg, Germany; ^3^Cognitive Science, Department of Humanities, Social and Political Sciences, Swiss Federal Institute of Technology in Zürich, Zürich, Switzerland; ^4^Department of Brain and Cognitive Engineering, Korea University, Seoul, South Korea

**Keywords:** wayfinding, strategy, spatial cognition, route knowledge, spatial representation, spatial learning, memory retrieval, memory load

## Abstract

Route selection is governed by various strategies which often allow minimizing the required memory capacity. Previous research showed that navigators primarily remember information at route decision points and at route turns, rather than at intersections which required straight walking. However, when actually navigating the route or indicating directional decisions, navigators make fewer errors when they are required to walk straight. This tradeoff between location memory and route decisions accuracy was interpreted as a “when in doubt follow your nose” strategy which allows navigators to only memorize turns and walk straight by default, thus considerably reducing the number of intersections to memorize. These findings were based on newly learned routes. In the present study, we show that such an asymmetry in route memory also prevails for planning routes within highly familiar environments. Participants planned route sequences between locations in their city of residency by pressing arrow keys on a keyboard. They tended to ignore straight walking intersections, but they ignored turns much less so. However, for reported intersections participants were quicker at indicating straight walking than turning. Together with results described in the literature, these findings suggest that a “when in doubt follow your nose strategy” is applied also within highly familiar spaces and might originate from limited working memory capacity during planning a route.

## INTRODUCTION

Navigating through the environment is crucial for many species to visit a known food source or to find the way back home. Typically, there are multiple ways to reach a destination, so navigators can apply several strategies to choose between alternative routes. Wayfinding strategies interact with the memory of an environment (Allen, 1999; Raubal and Worboys, 1999; Wiener et al., 2009). For example, in order to directly approach a non-visible goal via an unknown shortcut, metric “survey” knowledge about the environment is required. However, for deciding between familiar routes, such metric knowledge is not necessarily required (Wiener et al., 2009).

One strategy to reduce working memory load during route planning is to exploit hierarchical representations of an environment (Hirtle and Jonides, 1985; Richter et al., 2011) and apply fine-to-coarse route planning (Wiener and Mallot, 2003; Wiener et al., 2004). Within this stepwise strategy, navigators do not plan a route from A to B complete with all decisions in between, but rather plan the route first from A to an area in which B is located. As soon as navigators reach this target area, the remaining route is planned in detail. Consistent with such a strategy, navigators approach the target area as directly as possible.

Route strategies may not only reduce working memory load, but also long-term memory requirements. Sticking to a visible path, street, or corridor is a useful strategy within city or building environments (Allen, 1999). This strategy allows navigators to focus on decision points along their route while largely discarding further route information, as they will follow the path anyway. Consistent with such a strategy, objects located at decision points are mentioned more frequently and are recognized better than objects located elsewhere as shown in studies using familiar environments, newly learned virtual environments, routes displayed on a map, and routes presented via slides (Appleyard, 1969; Cohen and Schuepfer, 1980; Aginsky et al., 1997; Lee et al., 2002; Janzen, 2006).

This strategy of following a given path by default can unburden the required long-term memory by focusing on decision points. These requirements can be further reduced when applying a “when in doubt follow your nose” strategy, which is walking straight at decision points by default due to not knowing any better strategy. This strategy aids in remembering not all of the decision points, but only a subset of them (i.e., turns), thus further reducing the required memory while still being able to reach a goal. Computationally this approach is similar to dividing a route sequence by turns and chunking each turn with the straight walking before (Klippel et al., 2003; Richter and Klippel, 2005). Evidence for the “when in doubt follow your nose” strategy comes from an experiment asking participants to learn routes through a photorealistic, unknown virtual environment (Meilinger et al., 2012). Participants showed better memory for turns than for straight walking intersections as indicated by errors in drawing the routes. However, despite their better recall of turning intersections, they made fewer errors at straight walking intersections than at turning intersections when navigating the route or when indicating correct route continuation at randomly presented intersection pictures. The “when in doubt follow your nose” strategy explains these effects: participants focused on turning intersections and showed preference in remembering them. They did not remember straight walking intersections as well, but when asked for route decisions they more often reacted correctly at them, because walking straight was their default reaction.

Evidence for this “when in doubt follow your nose” strategy was obtained within an unfamiliar environment which consisted of single routes learned through video projection without motoric walking. It is an open question, whether such a strategy can also be observed within a highly familiar environment navigated in daily life, where routes are self-selected by the participants and not provided by the experimenter. In order to examine this question we reanalyzed data from another experiment (Meilinger et al., 2013). In this experiment, Tübingen residents were teleported to intersections within a photorealistic model of Tübingen, self-localized, and indicated the decision sequence along an imagined travel from their current location to a target location by pressing arrow keys on a keyboard. Within the present study, we compared errors along these route sequences between turning and straight walking intersections as well as the speed with which these route decisions were indicated. If the “when in doubt follow your nose” strategy is also prevalent within highly familiar environments we predicted there would be better recall for turning than for straight walking intersections. This is because if participants focus on turns they should forget them less often than straight walking. Additionally, for the remembered route decisions, we predicted quicker indication of straight walking than of turning. Straight walking is the default reaction and if executed, this execution should be easier than other reactions.

## MATERIALS AND METHODS

Twenty-three naïve participants (13 male) living in Tübingen for more than 2 years (M = 7.7; SD = 5.9), aged 18–50 years (M = 28.5; SD = 7.7) were recruited from a subject database and participated in exchange for monetary compensation after giving informed consent. They lived for at least 2 years in Tübingen (M = 7.7; SD = 5.9). The experiment was approved by the ethics committee of the University Clinics Tübingen.

**FIGURE 1:**
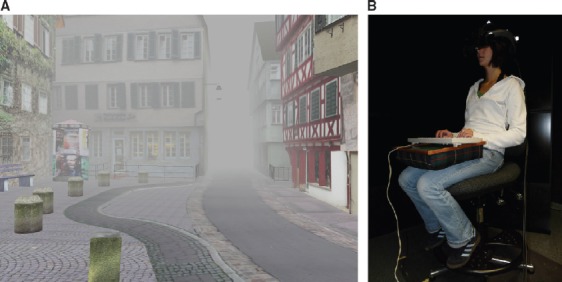
**Setup. (A)** A snapshot from Virtual Tübingen with fog hiding adjacent intersections. **(B)** A participant equipped with a HMD and a keyboard is typing in a route sequence. Reprinted from Meilinger et al. (2013), with permission from Elsevier.

We used Virtual Tübingen, a highly realistic virtual model of Tübingen, Germany (Figure 1; http://virtual.tuebingen.mpg.de; van Veen et al., 1998). Participants saw the model in horizontal perspective through a Kaiser SR80 head mounted display (HMD) while sitting on a swivel chair. Fog occluded adjacent intersections. We tracked head movements and rendered a stereo view of the virtual environment with a field of view of 63 (horizontal) × 53 (vertical) in real time. Participants typed in route sequences with the arrow keys of a custom keyboard resting on their legs (Figure 1). For further technical details regarding the setup please refer to Frankenstein et al. (2012).

In every trial, participants faced a start location, looked around, and confirmed recognition of location and orientation by pressing space. The written name of the target location (e.g., a tavern, train station, fire hall) appeared on the HMD-screen. To enter the route sequences leading to target locations, participants turned to face the initial direction of their chosen route, pressed the “up/forward” arrow key on the keyboard, entered the remaining sequence, and finished by pressing “space.” A sequence might look the following: “forward, left, right, forward, left.” Participants were explicitly told to report all navigation decisions (i.e., to include decisions to remain on the direction) at all intersections along the route, but to ignore gateways and dead-ends. Participants always remained at the starting location and were not moved through Virtual Tübingen, i.e., they faced the same scene as if they were standing at that start location during the whole recall procedure. Participants controlled inter-trial intervals themselves, and did not receive any feedback. They had successfully identified start and target locations on snapshots displaying only locally visible landmarks before the experiment. Start and target locations encompassed commonly known locations within Tübingen. They were chosen to cover multi-alternative, overlapping routes. All routes started within the area covered by Virtual Tübingen, but targets came from a larger area. For a map of start and target locations please refer to Frankenstein et al. (2012).

We tested participants’ spatial memory in two imagined perspectives, every participant entered each sequence twice. In the *walk* perspective, participants were asked to indicate route sequences as if they were walking this route (i.e., in ground perspective). In the *bird* perspective, participants were asked to imagine looking down on the city and report the route as if looking on a map (i.e., include terms like “up” and “down” on the map). This variation was used to infer the reference frames underlying route memory in the previous study. The observed advantage for reporting from walk perspective indicates that participants represented the routes not within a single reference frame as within a map, but most likely along multiple, local reference frames (Meilinger et al., 2013).

Participants performed two blocks of 30 trials each in walk and bird perspective with the order of perspectives counterbalanced between participants. Within a block the progression of trials was the following: participants were teleported to a start location, indicted the routes to several target locations (seven targets for two of the four start locations, and eight targets on the other two start locations) and then proceeded to the next start location. The order of start locations and target locations at a single start location were chosen randomly for each participant and block.

After the experiment, participants indicated the routes entered before into paper maps of Tübingen. For every participant, his/her individual drawn routes were used as the reference relative to determine the individual errors in the entered sequences. Reported errors compared the number of required turns, or straight walking, with how often such commands were entered. Errors were omissions (coded as negative errors, e.g., entering fewer turns as required), insertions (coded as positive errors, e.g., entering more turns as required) and wrong turning direction (i.e., too many left or right turns). Turning direction did not show any effect and is irrelevant for the current analysis. Deviations from required straights and turns do not consider errors in route order, for example, typing left–right instead of right–left. Levenshtein or “edit” distance (Levenshtein, 1966) does so to some extent: it estimates the minimum number of sequence elements to be altered, inserted, or erased in order to obtain the reference sequence from the entered sequence. Usually, different possibilities of alteration exist, so errors are still difficult to attribute to individual intersections. Analyzing our data by Levenshtein distances vs. the absolute number of errors (Levenshtein distances do not consider over- vs. underestimation), we observed very similar effects. We thus conclude that route order errors were not central.

For the entered route decisions, we compared latencies between turns and straight walking. Trials with error or latency data deviating more than three standard deviations from the overall mean and trials with wrongly mapped starting or goal locations within the maps were excluded from analysis (13%, i.e., 186 trials). Participants’ individual error or latency means were submitted to an ANOVA, with the within participant factors “intersection” (straight vs. turns) and “perspective” (walk vs. bird), as well as the between-participants factor “perspective order” (bird first vs. walk first). There were no gender differences, therefore, pooled data is reported.

## RESULTS

**FIGURE 2:**
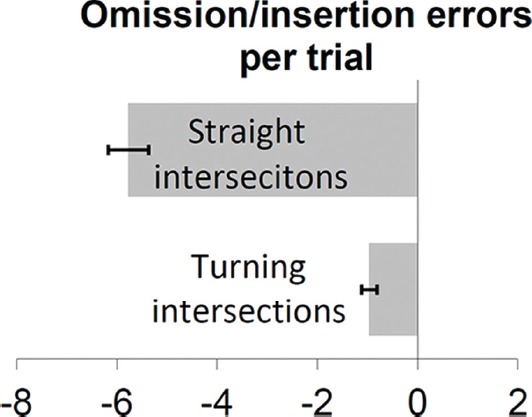
**Omission errors (negative numbers) and insertion errors (positive numbers) per trial at turning and straight walking intersections.** Error bars indicate standard errors of the mean as estimated from the marginal means.

Errors in route recall mainly consisted of omission errors. Participants on average missed 6.7 or 45% of the intersections per trial. Entered sequences were shorter than correct sequences, *t*(22) = 14.6, *p* < 0.001. As shown in Figure 2, forgetting differed substantially between the types of intersections. Participants on average missed 5.77 or 60% straight walking intersections per trial, but only 0.96 or 23% turns. This was clearly less often, *F*(1,21) = 135, *p* < 0.001, *η*^2^ = 0.87, even when analyzing percent instead of number of omissions, *F*(1,21) = 53.1, *p* < 0.001, *η*^2^ = 0.72. Consistent with a “when in doubt follow your nose” strategy, this suggests that participants preferably focused on turning intersections also in highly familiar environments.

The difference in errors (turns vs. straight) was larger in the bird perspective than in the walking perspective as indicated by the interaction perspective × intersection type, *F*(1,21) = 15.11, *p* = 0.001, $\eta_{p}^{2}$ = 0.42. Participants forgot more straight than turn intersections in bird perspective, *F*(1,21) = 15.6, *p* = 0.001, $\eta_{p}^{2}$ = 0.43, which was less pronounced and only indicated by a trend in walk perspective, *F*(1,21) = 3.04, *p* = 0.096, $\eta_{p}^{2}$ = 0.13. The general advantage in reporting from walk perspective is discussed elsewhere (Meilinger et al., 2013). Please note that participants on average entered straight walking 3.6 times per route which is roughly comparable to the 3.2 turns per route, *F*(1,21) = 1.44, *p* = 0.243, $\eta_{p}^{2}$ = 0.06. As the selected routes typically required more straight walking decisions the errors in straight walking were higher.

Prior evidence for the “when in doubt follow your nose” strategy showed not only diminished recall of straight walking intersections, but also better performance when indicating straight walking as compared to turning. The same pattern is also shown in the present data. There was a general trend for participants to more quickly indicate straight walking as compared with turning, *F*(1,21) = 3.00, *p* = 0.10, $\eta_{p}^{2}$ = 0.12, which was significant for walk perspective, *F*(1,21) = 7.41, *p* = 0.012, $\eta_{p}^{2}$ = 0.25, but not bird perspective, F < 1 (see Table 1). There was no interaction between perspective and intersection type, *F*(1,21) = 2.31, *p* = 0.14, $\eta_{p}^{2}$ = 0.10.

**Table 1:** **Means and between participant standard errors in seconds for indicating straight walking and turning as a function of test perspective**.

**Intersection type**	**Walk perspective**		**Bird perspective**	
	*M*	*SE*	*M*	*SE*
Straight	2.41	0.22	2.52	0.22
Turn	2.60	0.21	2.55	0.20

## DISCUSSION

Participants mainly forgot to indicate straight walking as compared with turns, but for the reported intersections participants were quicker at indicating straight walking than turns. Related work examining newly learned routes found the same tradeoff pattern of better recall for turns, but higher performance for indication straight walking at an intersection (Meilinger et al., 2012). This pattern is consistent with the “when in doubt follow your nose” strategy first described for exploring novel environments by Dalton (2003), and applied to route memory by Meilinger et al. (2012). This strategy proposes that participants by default walk straight. Therefore, they are not required to memorize straight walking intersections and can only memorize turns, thus reducing memory load. Consequently, navigators will report more turns than straight walking intersections. However, when indicating route decisions, the default strategy of walking straight will facilitate the indicating of straight walking as this is the behavioral default and thus more easily executed. While this pattern was shown before for learning unfamiliar routes, the present results extend these findings toward a highly familiar environment, from visually learning by video toward learning by navigating the real world, and from route learning to route planning. The “when in doubt follow your nose” strategy seems to be applied in a wide variety of situations.

The higher omission rate, but quicker reactions for straight walking as compared with turns was qualified by the perspective from which the task was conducted. On the one hand, omission rate difference between intersections was larger when participants answered from an imagined bird perspective. On the other hand, quicker reactions for straight walking were only found in walk perspective. Again, this parallels results from the prior route learning study where higher omission rates for straight walking were found in a bird’s eye perspective task (i.e., map drawing), but not a horizontal task (i.e., recognizing intersection pictures). In contrast, better performance in route continuation at straight intersections was observed in walk perspective tasks (i.e., in navigating the route and in indicating route continuation from intersection pictures) although route continuation was not tested from bird perspective. From our point of view, the dependence on walk perspective for the better performance in indicating straight route continuations vs. turns, originates from body based reference frames. Default reactions executed more often can be performed quicker or more accurately than other reactions (Anderson, 2000). For the case of navigating straight this is body-relative. It can be expressed by walking straight, flexing a joystick to the front, or by pressing the straight ahead/up arrow key, yielding advantages observed within the two experiments. However, when answering from bird perspective straight ahead meant keeping the same walking direction which most often did not coincide with bodily front. After turning eastward, for example, by pressing the right arrow key, straight walking had to be indicated by pressing the right arrow key again as this was the same direction in bird perspective. This was not along the bodily front and we speculate that this was the reason why we did not observe a straight walking advantage in that perspective. Support for the advantage of bodily front comes from pointing experiments where there is an observed advantage of pointing to the bodily front as compared with the pointing to the back (Sholl, 1987, 1999). Future experimentation has to clarify this issue.

Omission errors occur more frequently at straight than at turn intersections. This difference was stronger in bird perspective than in walk perspective. We are hesitant to state that the difference is only to be found in bird perspective. In walk perspective there was a trend for more omission errors as well. While a trend does not qualify as a statistical reliable difference, it is also no argument to suggest that there is no difference at all. From our point of view it is quite possible that power was not sufficient to find a smaller effect in walk perspective. Nevertheless, the significant interaction suggests that the effect was clearly stronger in the bird than in walk perspective. Why would this be the case? We think we can exclude the possibility that participants used map-based knowledge in the vertical perspective, and navigation based knowledge in the walk perspective. The prior data analysis of the same data used here (Meilinger et al., 2013) suggests that route errors and latencies from both perspectives were completely unrelated to map-acquired pointing errors in parallelized pointing tasks. Such a correlation should have been present if route testing from bird perspective was map-based. Together with the observed advantage in answering from walk perspective vs. bird perspective the data suggests that participants’ route sequences were derived from multiple local reference frames likely acquired from navigation. When answering from bird perspective, participants had to transform these reference frames into bird perspective first. This transformation puts an additional load on participants. As pointed out in the following paragraphs we think that the preference for turns results from limited working memory. Additional working memory load due to perspective transformations will then foster the observed concentration on turns observed in the interaction.

In learning unfamiliar routes, the “when in doubt follow your nose” strategy affected long-term memory storage (Meilinger et al., 2012). Turning intersections were recalled more accurately than straight walking intersections. In the present study, better memory for turning intersections might have originated from two sources: better long-term memory for turns or alternatively loading preferable turns from long-term memory into working memory when selecting the route to the goal. We cannot differentiate between these alternatives based on the given data. However, in the light of other work, the interpretation on working memory seems more plausible as pointed out in the following.

One argument that long-term memory does not primarily consist of turns states that within ones city of residency all intersections can be sufficiently familiar. Long-term memory is not considered as limited in capacity (Anderson, 2000). With sufficient training, large information sets can be learned and our long-term inhabitants had years of daily experience to learn the whole environment. While it might not make sense to learn every driveway, gateway, dead-end, or small back road, exactly these were excluded in the present study as participants only focused on streets providing an alternative route to the goal. Furthermore, participants chose routes themselves, so one can expect them to only have selected familiar routes and prior testing ensured familiarity with the start and goal locations before the experiment. All these points suggest that participants could in principle be familiar with all intersections, not just a subset of them.

Another argument against long-term memory priority for turns follows from the structure of route memory. It is highly unlikely that participants memorized only individual routes and selected the route from that pool. The number of potential routes through a city is just too large to memorize them one by one. Consequently, models of route knowledge consider networks or graphs of locations connected via paths from which routes can be selected flexibly (Poucet, 1993; Trullier et al., 1997; Werner et al., 2000; Meilinger, 2008; Mallot and Basten, 2009). These conceptions were also supported empirically (Chrastil and Warren, 2014). However, within such a graph, one and the same intersection can be a turn on one route, but a straight walking intersection on another one. Omitting memory for one intersection may omit a straight walking intersection on one route, but a turning intersection at another route also within the present experiment. Consequently, a decision point within a network cannot be assigned to turning or straight walking. It is, therefore, not very plausible that straight walking intersections were not present in long-term memory.

Omitting straight walking intersections from long-term memory is not plausible. However, omitting them from working memory during route planning is plausible. Working memory capacity is limited (Anderson, 2000), therefore, concentrating on turns can reduce memory load. This can be especially important when the working memory content has to be transformed. As pointed out before, testing from bird perspective involved transforming intersections from walk perspective (i.e., the format of long-term storage) into bird perspective within working memory. Such additional working memory load will enhance the concentration on turns compared with testing from walk perspective where no additional load is present. Working memory limitations can thus explain the observed perspective differences. Limited working memory resources can be interpreted as attentional focus (Kane and Engle, 2002). In that sense participants only attended a subset of intersections during planning—the ones within working memory. The “when in doubt follow your nose” strategy allowed navigators to limit this focus on turns. Please note, there is hardly any “doubt” at an intersection when planning a route. It is more the question of which intersections are attended in working memory rather than about if all intersections are considered and some are doubted. One might argue that participants are not required to transfer anything into working memory during planning as they can directly give an answer at an intersection. However, this seems wrong as they first have to select a route to the goal (or at least the goal area) before being able to enter route decisions. Otherwise they would have entered random sequences which they clearly did not do. Please note that classical search strategies through a (problem) space also assume limited working memory and some of them minimize exactly that load (e.g., iterative deep search; Russell and Norvig, 1995).

Reducing working memory load is also the basis for other wayfinding strategies. Fine-to-coarse planning (Wiener and Mallot, 2003) does so by planning not the full route, but only the route toward the goal area first. When reaching the goal area, the previous route can be discarded from working memory and the remaining route within the goal area to the target is planned. The working memory interpretation of the “when in doubt follow your nose” strategy would draw on exactly the same underlying reason. This line of argumentation also fits for the strategy of minimizing turns (Golledge, 1995). If the “when in doubt follow your nose” strategy reduces working memory by only representing turns, memory load can be even further reduced when selecting a route which consists of as few turns as possible.^1^ In that sense all three wayfinding strategies would be grounded in the more general principle of reducing working memory load. Please note that these strategies can be applied at the same time.

In summary, we conclude that the higher accuracy of recalling turning intersections is grounded in working memory. This conclusion originates from several arguments: (1) within ones city of residency all intersections—straight walking and turns—can be equally familiar, (2) intersections within long-term route memory are hardly classified as a turn or straight walking, (3) working memory during planning is necessarily capacity limited and the “when in doubt follow your nose” strategy can reduce this load, and (4) other wayfinding strategies also limit working memory load.

The present pattern was found for planning routes but not for navigating routes. Hölscher et al. (2011) have shown that the selected routes when planning before navigation differed from the routes chosen when actually navigating a route. The “when in doubt follow your nose” strategy was examined in actual navigation (Meilinger et al., 2012), however, it was a situation where paths were given and navigators could not choose their route alternative. As a consequence we do not know whether results apply to free navigation within a familiar environment. Participants might swap to a applying a least angle strategy when actually moving through the environment rather than follow the selected route. Swapping might be more difficult within Tübingen which has an irregular street pattern as compared to the center of Freiburg in the Hölscher et al. (2011) study which roughly follows a grid pattern making the application of the observed least angle strategy considerably easier.

Can the higher accuracy in recalling turns be explained by participants misunderstanding the task, thinking that they are asked to only report turns? This seems unlikely as we explicitly instructed participants to report all intersections including the straight walking ones. Furthermore, such misunderstanding would only be plausible if route memory indeed focuses on turns rather than straight walking intersections, which is exactly what the “when in doubt follow your nose” strategy states.

Participants showed a lot of omission errors. Even if considering only omission errors of required turns, participants forgot to enter one turn per route on average. Does this mean that participants are not able to find routes within their city of residency? We do not think so. The present experiment differed in various aspects from real navigation. First, decisions were time pressured. In real life, one can wait and think about the next decision which will minimize errors. Furthermore, for half of the routes, participants recalled the routes from a bird’s eye perspective not encountered within real life. Transforming the route knowledge into this unusual perspective likely increased the error rate. Most importantly, participants recalled the routes while remaining at the start location, i.e., they entered the decisions without any visual cues except for the start location. Memory performance with visual cues (e.g., recognition) is typically much better than without cues (i.e., recall; Anderson, 2000). Consequently, participants can be expected to perform much better in real life than within the present experimental setting.

Just as with newly learned routes, the present results on planning routes within a familiar space are consistent with a “when in doubt follow your nose” strategy. Several arguments suggest that the effect in planning is due to limiting working memory load—a general principle which can account for several wayfinding strategies.

## AUTHOR CONTRIBUTIONS

Tobias Meilinger, Julia Frankenstein, and Heinrich H. Bülthoff designed the work and wrote the paper. Tobias Meilinger and Julia Frankenstein collected and analyzed the data.

### Conflict of Interest Statement

The authors declare that the research was conducted in the absence of any commercial or financial relationships that could be construed as a potential conflict of interest.

## Footnotes

1 The defer path choice strategy (Christenfeld, 1995) which avoids turns as long as possible or the longest initial segment strategy (Bailenson et al., 2000) of trying to approach the goal with the longest possible straight route might be instances of the minimizing turn strategy—although these strategies might in the end not always select the route with the fewest turns.
